# μ_2_-*m*-Xylylenebis(salicylaldiminato)-bis­(η^4^-1,5-cyclo­octa­diene)dirhodium(I) dichloro­methane solvate

**DOI:** 10.1107/S1600536812040603

**Published:** 2012-09-29

**Authors:** Stacie Gregory, Ravi K. Laxman, Frank R. Fronczek, Andrew W. Maverick, Steven F. Watkins

**Affiliations:** a Department of Chemistry, Louisiana State University, Baton Rouge LA 70803-1804 USA

## Abstract

In the title solvate, [Rh_2_(C_22_H_18_N_2_O_2_)(C_8_H_12_)_2_]·CH_2_Cl_2_, each organometallic mol­ecule is composed of two Rh^I^ cations, the tetra­dentate dianion α,α′-bis­(salicylaldiminato)-*m*-xylene and two 1,5-cyclo­octa­diene (COD) ligands. Each Rh^I^ atom is coordinated by one O atom [Rh—O = 2.044 (2) and 2.026 (2) Å], one N atom [Rh—N = 2.083 (2) and 2.090 (2) Å], and one COD ligand *via* two η^2^-bonds, each directed toward the mid-point of a C=C bond (*Cg*): Rh—*Cg* = 2.007 (2), 2.013 (2), 2.000 (2) and 2.021 (2) Å. Each Rh^I^ atom has a quasi-square-planar coordination geometry, with average r.m.s. deviations of 0.159 (1) and 0.204 (1) Å from the mean planes defined by Rh and the termini of its four coordinating bonds. The two COD ligands have quasi-*C*_2_ symmetry, twisted from ideal *C*_2*v*_ symmetry by 30.0 (3) and −33.1 (3)°, and are quasi-enanti­omers of one another. The intra­molecular Rh⋯Rh distance of 5.9432 (3) Å suggests that there is no direct metal–metal inter­action.

## Related literature

For related structures, see: Mosae Selvakumar *et al.* (2011 ▶); Maverick *et al.* (2005 ▶); Nakamura *et al.* (2001 ▶). For the synthesis, see: Brunner & Fisch (1987 ▶). For the Universal Force Field procedure, see: Rappe *et al.* (1992 ▶).
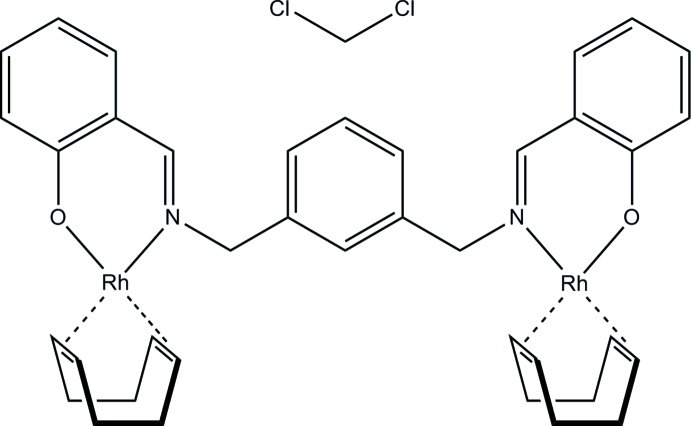


## Experimental

### 

#### Crystal data

[Rh_2_(C_22_H_18_N_2_O_2_)(C_8_H_12_)_2_]·CH_2_Cl_2_
*M* *_r_* = 849.48
Monoclinic, 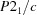

*a* = 16.0829 (4) Å
*b* = 18.8607 (4) Å
*c* = 11.2352 (2) Å
β = 99.081 (1)°
*V* = 3365.31 (13) Å^3^
*Z* = 4
Mo *K*α radiation
μ = 1.18 mm^−1^
*T* = 90 K
0.30 × 0.12 × 0.12 mm

#### Data collection

Nonius KappaCCD diffractometer
Absorption correction: multi-scan (*HKL* *SCALEPACK*; Otwinowski & Minor 1997 ▶) *T*_min_ = 0.631, *T*_max_ = 0.912
17250 measured reflections
10328 independent reflections
8682 reflections with *I* > 2σ(*I*)
*R*_int_ = 0.025

#### Refinement

*R*[*F* ^2^ > 2σ(*F* ^2^)] = 0.032
*wR*(*F* ^2^) = 0.077
*S* = 1.02
10328 reflections
425 parameters
H-atom parameters constrained
Δρ_max_ = 0.93 e Å^−3^
Δρ_min_ = −0.96 e Å^−3^

### 

Data collection: *COLLECT* (Nonius, 2000 ▶); cell refinement: *HKL* *SCALEPACK* (Otwinowski & Minor, 1997 ▶); data reduction: *HKL* *DENZO* (Otwinowski & Minor, 1997 ▶) and *SCALEPACK*; program(s) used to solve structure: *SHELXS97* (Sheldrick, 2008 ▶); program(s) used to refine structure: *SHELXL97* (Sheldrick, 2008 ▶); molecular graphics: *ORTEP-3 for Windows* (Farrugia, 1997 ▶); software used to prepare material for publication: *WinGX* (Farrugia, 1999 ▶) and *GAUSSIAN09* (Frisch *et al.*, 2009 ▶).

## Supplementary Material

Crystal structure: contains datablock(s) global, I. DOI: 10.1107/S1600536812040603/cv5334sup1.cif

Structure factors: contains datablock(s) I. DOI: 10.1107/S1600536812040603/cv5334Isup2.hkl

Additional supplementary materials:  crystallographic information; 3D view; checkCIF report

## Figures and Tables

**Fig. 1.**
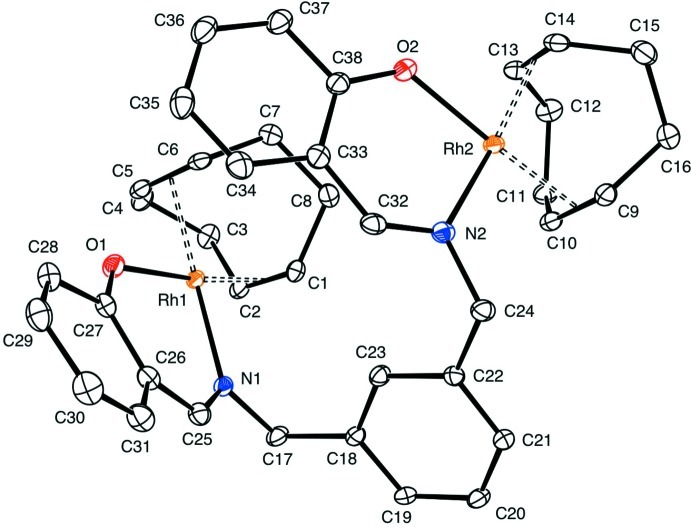
View of (I) showing the atomic labeling and 50% probability displacement ellipsoids. The solvent molecule and H atoms were omitted for clarity.

## supplementary crystallographic information

### Comment

Schiff-base ligands such as SIXH_2_ (C_22_H_20_N_2_O_2_, CAS 51540–97–7, Maverick *et al.*, 2005) are typically formed by condensation of a primary amine with an aldehyde, resulting in an imine, *R*^1^HC=N—*R*^2^, which is particularly useful for binding metal ions. The title compound, (SIX)Rh_2_(COD)_2_.DCM, C_38_H_42_N_2_O_2_Rh_2_.CH_2_Cl_2_, consists of one DCM molecule for each organometallic dimer. The latter is composed of tetradentate dianion α,α'-bis(salicylimino)-*m*-xylene (SIX) coordinated to two Rh(I) ions. Each Rh is bonded to an oxygen (Rh1—O1 = 2.044 (2), Rh2—O2 = 2.026 (2) Å), a nitrogen (Rh1—N1 = 2.083 (2), Rh2—N2 = 2.090 (2) Å), and a 1,5-cyclooctadiene (COD) ligand *via* two η^2^-bonds (each directed toward the center of a C═C bond): Rh1—(C1 & C2) = 2.007 (2), Rh1— (C5 & C6) = 2.013 (2), Rh2—(C9 & C10) = 2.000 (2), Rh2—(C13 & C14) = 2.021 (2) Å.

Each COD has quasi-C_2_ molecular symmetry as judged by close equivalencies of putatively equal intraannular bond lengths, bond angles and torsion angles. One measure of the twist of each ring from idealized C_2v_ molecular symmetry is the torsion angle of four atom-pair centroids: C1 & C2, C3 & C8, C4 & C7, C5 & C6 (+30.0 (3)°) and C9 & C10, C11 & C16, C12 & C15, C13 & C14 (-33.1 (3)°). The signs of the two twist angles, together with the signs of equivalent intraannular torsion angles, indicates that the two COD moieties are near enantiomorphs. The observed molecular measurements are very close to those for the minimum energy conformer calculated in Gaussian09 (Frisch *et al.*, 2009) using the UFF procedure (Rappe *et al.*, 1992). This conformer has exact C_2_ symmetry and twist angle ±30.8°.

The four bonds about each Rh form a quasi-square planar environment. For example, deviations δ_r.m.s._ from the mean planes defined by each metal atom and the termini of its four bonds are Rh1: 0.159 (1) and Rh2: 0.204 (1) Å. The intramolecular Rh···Rh distance of 5.943 (1) Å suggests that there is no direct metal-metal interaction.

### Experimental

The synthesis of (SIX)Rh_2_(COD)_2_ was similar to that employed for (salicylimino)Rh(COD) complexes (Brunner & Fisch, 1987). Rh_2_(COD)_2_Cl_2_ was suspended in diethyl ether and a stoichiometric amount of solid SIXH_2_ (Maverick *et al.*, 2005) was added. The mixture was cooled to 0 °C, a slight excess of 1*M* NaOH(aq) was added, and the mixture stirred for 1 h. The organic layer was dried over anhydrous NaSO_4_, filtered, and the solvent was removed to give bright yellow flaky solid. Crystals were obtained by slow evaporation from dichloromethane.

### Refinement

All H atoms were placed in calculated positions, guided by difference maps, with C—H bond distances 0.95 (C*sp*^2^) and 0.99 (C*sp*^3^) Å, and *U*_iso_=1.2*U*_eq_, thereafter refined as riding.

### Crystal data

**Table:**

[Rh_2_(C_22_H_18_N_2_O_2_)(C_8_H_12_)_2_]·CH_2_Cl_2_	*F*(000) = 1728
*M**_r_* = 849.48	*D*_x_ = 1.677 Mg m^−^^3^
Monoclinic, *P*2_1_/*c*	Mo *K*α radiation, λ = 0.71073 Å
Hall symbol: -P 2ybc	Cell parameters from 9881 reflections
*a* = 16.0829 (4) Å	θ = 2.6–31.0°
*b* = 18.8607 (4) Å	µ = 1.18 mm^−^^1^
*c* = 11.2352 (2) Å	*T* = 90 K
β = 99.081 (1)°	Lath, yellow
*V* = 3365.31 (13) Å^3^	0.30 × 0.12 × 0.12 mm
*Z* = 4	

### Data collection

**Table:**

Nonius KappaCCD diffractometer	10328 independent reflections
Radiation source: Enraf Nonius FR590	8682 reflections with *I* > 2σ(*I*)
Horizonally mounted graphite crystal monochromator	*R*_int_ = 0.025
Detector resolution: 9 pixels mm^-1^	θ_max_ = 30.8°, θ_min_ = 2.6°
CCD rotation images, thick slices scans	*h* = −23→23
Absorption correction: multi-scan (*HKL**SCALEPACK*; Otwinowski & Minor 1997)	*k* = −27→20
*T*_min_ = 0.631, *T*_max_ = 0.912	*l* = −15→15
17250 measured reflections	

### Refinement

**Table:**

Refinement on *F*^2^	Secondary atom site location: difference Fourier map
Least-squares matrix: full	Hydrogen site location: inferred from neighbouring sites
*R*[*F*^2^ > 2σ(*F*^2^)] = 0.032	H-atom parameters constrained
*wR*(*F*^2^) = 0.077	*w* = 1/[σ^2^(*F*_o_^2^) + (0.0322*P*)^2^ + 4.6131*P*] where *P* = (*F*_o_^2^ + 2*F*_c_^2^)/3
*S* = 1.02	(Δ/σ)_max_ = 0.003
10328 reflections	Δρ_max_ = 0.93 e Å^−^^3^
425 parameters	Δρ_min_ = −0.96 e Å^−^^3^
0 restraints	Extinction correction: *SHELXL97* (Sheldrick, 2008), Fc^*^=kFc[1+0.001xFc^2^λ^3^/sin(2θ)]^-1/4^
0 constraints	Extinction coefficient: 0.00049 (10)
Primary atom site location: structure-invariant direct methods	

### Special details

**Table:**

Geometry. All e.s.d.'s (except the e.s.d. in the dihedral angle between two l.s. planes) are estimated using the full covariance matrix. The cell e.s.d.'s are taken into account individually in the estimation of e.s.d.'s in distances, angles and torsion angles; correlations between e.s.d.'s in cell parameters are only used when they are defined by crystal symmetry. An approximate (isotropic) treatment of cell e.s.d.'s is used for estimating e.s.d.'s involving l.s. planes.
Refinement. Refinement of *F*^2^ against ALL reflections. The weighted *R*-factor *wR* and goodness of fit *S* are based on *F*^2^, conventional *R*-factors *R* are based on *F*, with *F* set to zero for negative *F*^2^. The threshold expression of *F*^2^ > σ(*F*^2^) is used only for calculating *R*-factors(gt) *etc*. and is not relevant to the choice of reflections for refinement. *R*-factors based on *F*^2^ are statistically about twice as large as those based on *F*, and *R*-factors based on ALL data will be even larger.

### Fractional atomic coordinates and isotropic or equivalent isotropic displacement parameters (Å^2^)

**Table:**

	*x*	*y*	*z*	*U*_iso_*/*U*_eq_	
C1	0.32235 (14)	0.67047 (12)	0.42132 (19)	0.0144 (4)	
H1	0.316	0.6493	0.4961	0.017*	
C2	0.40469 (14)	0.68544 (12)	0.40159 (19)	0.0144 (4)	
H2	0.4504	0.6714	0.4611	0.017*	
C3	0.42401 (15)	0.72324 (13)	0.2890 (2)	0.0180 (4)	
H3A	0.4744	0.7535	0.3111	0.022*	
H3B	0.3761	0.7545	0.2573	0.022*	
C4	0.43978 (15)	0.67113 (13)	0.1897 (2)	0.0183 (4)	
H4A	0.4226	0.6936	0.11	0.022*	
H4B	0.5008	0.6608	0.1986	0.022*	
C5	0.39205 (15)	0.60237 (12)	0.19427 (18)	0.0147 (4)	
H5	0.4224	0.5594	0.1911	0.018*	
C6	0.30668 (15)	0.59744 (12)	0.20276 (18)	0.0150 (4)	
H6	0.2832	0.5516	0.2085	0.018*	
C7	0.24899 (15)	0.66143 (13)	0.2033 (2)	0.0181 (4)	
H7A	0.1921	0.6492	0.1608	0.022*	
H7B	0.2709	0.701	0.1594	0.022*	
C8	0.24272 (14)	0.68541 (13)	0.3328 (2)	0.0179 (4)	
H8A	0.2309	0.7369	0.3325	0.022*	
H8B	0.1948	0.6607	0.3602	0.022*	
C9	−0.03645 (14)	0.67669 (13)	0.53838 (19)	0.0156 (4)	
H9	−0.04	0.6301	0.5695	0.019*	
C10	0.04403 (14)	0.70502 (12)	0.53404 (19)	0.0148 (4)	
H10	0.0915	0.6772	0.5666	0.018*	
C11	0.06048 (15)	0.77664 (13)	0.4812 (2)	0.0169 (4)	
H11A	0.1204	0.7795	0.471	0.02*	
H11B	0.0499	0.8143	0.5383	0.02*	
C12	0.00494 (15)	0.79022 (13)	0.3585 (2)	0.0192 (4)	
H12A	−0.0482	0.8132	0.3718	0.023*	
H12B	0.0345	0.823	0.3105	0.023*	
C13	−0.01528 (15)	0.72200 (13)	0.28883 (19)	0.0169 (4)	
H13	0.0242	0.7059	0.2402	0.02*	
C14	−0.08689 (14)	0.68111 (13)	0.2903 (2)	0.0168 (4)	
H14	−0.0936	0.6403	0.2402	0.02*	
C15	−0.15524 (15)	0.69641 (14)	0.3656 (2)	0.0206 (5)	
H15A	−0.1913	0.6539	0.3666	0.025*	
H15B	−0.191	0.7357	0.3283	0.025*	
C16	−0.11809 (15)	0.71664 (14)	0.4959 (2)	0.0198 (5)	
H16A	−0.1071	0.7683	0.5004	0.024*	
H16B	−0.1594	0.7055	0.5498	0.024*	
C17	0.40803 (14)	0.58867 (13)	0.65856 (19)	0.0156 (4)	
H17A	0.4215	0.6372	0.6339	0.019*	
H17B	0.4547	0.5725	0.7207	0.019*	
C18	0.32686 (14)	0.59036 (12)	0.71220 (18)	0.0128 (4)	
C19	0.32798 (14)	0.61215 (12)	0.83111 (19)	0.0149 (4)	
H19	0.3792	0.6278	0.8777	0.018*	
C20	0.25437 (15)	0.61109 (13)	0.88182 (19)	0.0175 (4)	
H20	0.2555	0.6261	0.9628	0.021*	
C21	0.17942 (14)	0.58825 (12)	0.81466 (19)	0.0158 (4)	
H21	0.1298	0.5863	0.8506	0.019*	
C22	0.17638 (14)	0.56802 (12)	0.69440 (19)	0.0133 (4)	
C23	0.25022 (14)	0.56963 (12)	0.64469 (19)	0.0136 (4)	
H23	0.2485	0.5563	0.5628	0.016*	
C24	0.09392 (14)	0.54057 (13)	0.62580 (19)	0.0157 (4)	
H24A	0.0882	0.4898	0.6456	0.019*	
H24B	0.047	0.5664	0.6537	0.019*	
C25	0.40126 (14)	0.47439 (13)	0.5820 (2)	0.0157 (4)	
H25	0.4112	0.4648	0.6661	0.019*	
C26	0.38851 (14)	0.41316 (12)	0.5056 (2)	0.0154 (4)	
C27	0.38728 (13)	0.41557 (12)	0.3777 (2)	0.0144 (4)	
C28	0.38042 (15)	0.34953 (13)	0.3159 (2)	0.0181 (4)	
H28	0.3838	0.3488	0.2323	0.022*	
C29	0.36906 (15)	0.28658 (13)	0.3732 (2)	0.0206 (5)	
H29	0.3636	0.2437	0.3283	0.025*	
C30	0.36537 (16)	0.28506 (13)	0.4979 (2)	0.0221 (5)	
H30	0.3554	0.2419	0.537	0.027*	
C31	0.37645 (15)	0.34719 (13)	0.5613 (2)	0.0193 (4)	
H31	0.3761	0.3462	0.6457	0.023*	
C32	0.12119 (14)	0.49715 (12)	0.4418 (2)	0.0157 (4)	
H32	0.148	0.4618	0.4947	0.019*	
C33	0.12575 (14)	0.48665 (12)	0.3159 (2)	0.0157 (4)	
C34	0.17486 (15)	0.42879 (13)	0.2857 (2)	0.0193 (4)	
H34	0.204	0.4004	0.3487	0.023*	
C35	0.18162 (16)	0.41255 (13)	0.1683 (2)	0.0215 (5)	
H35	0.215	0.3736	0.1504	0.026*	
C36	0.13822 (16)	0.45477 (14)	0.0750 (2)	0.0219 (5)	
H36	0.1419	0.4438	−0.0065	0.026*	
C37	0.09040 (15)	0.51187 (13)	0.1008 (2)	0.0191 (4)	
H37	0.0624	0.54	0.0366	0.023*	
C38	0.08211 (14)	0.52949 (13)	0.22172 (19)	0.0161 (4)	
N1	0.40120 (11)	0.54087 (10)	0.55282 (16)	0.0130 (3)	
N2	0.08572 (12)	0.54799 (11)	0.49311 (16)	0.0144 (3)	
O1	0.39194 (10)	0.47352 (9)	0.31685 (13)	0.0155 (3)	
O2	0.03491 (11)	0.58322 (9)	0.23851 (14)	0.0174 (3)	
Rh1	0.379269 (10)	0.575178 (9)	0.374373 (14)	0.01104 (5)	
Rh2	0.017893 (10)	0.629409 (9)	0.395686 (14)	0.01219 (5)	
C39	0.35225 (17)	0.44841 (14)	−0.0064 (2)	0.0236 (5)	
H39A	0.3258	0.4464	0.0673	0.028*	
H39B	0.3566	0.4988	−0.0291	0.028*	
Cl1	0.28848 (5)	0.40257 (4)	−0.12430 (7)	0.03484 (16)	
Cl2	0.45414 (4)	0.41086 (4)	0.02361 (6)	0.03307 (16)	

### Atomic displacement parameters (Å^2^)

**Table:**

	*U*^11^	*U*^22^	*U*^33^	*U*^12^	*U*^13^	*U*^23^
C1	0.0170 (10)	0.0127 (10)	0.0140 (9)	0.0003 (8)	0.0040 (8)	−0.0027 (8)
C2	0.0174 (10)	0.0132 (10)	0.0127 (9)	0.0001 (8)	0.0029 (8)	−0.0017 (8)
C3	0.0198 (11)	0.0155 (10)	0.0195 (11)	−0.0041 (9)	0.0057 (8)	−0.0009 (8)
C4	0.0216 (11)	0.0188 (11)	0.0159 (10)	−0.0029 (9)	0.0076 (8)	−0.0007 (8)
C5	0.0202 (11)	0.0146 (10)	0.0091 (9)	0.0011 (8)	0.0018 (8)	−0.0006 (8)
C6	0.0200 (11)	0.0153 (10)	0.0089 (9)	0.0004 (8)	−0.0009 (8)	0.0009 (8)
C7	0.0166 (10)	0.0201 (11)	0.0167 (10)	0.0016 (9)	0.0004 (8)	0.0018 (9)
C8	0.0162 (10)	0.0188 (11)	0.0192 (10)	0.0017 (9)	0.0040 (8)	−0.0001 (9)
C9	0.0167 (10)	0.0175 (11)	0.0127 (9)	−0.0003 (8)	0.0030 (8)	−0.0005 (8)
C10	0.0160 (10)	0.0174 (10)	0.0108 (9)	0.0000 (8)	0.0015 (7)	−0.0008 (8)
C11	0.0172 (10)	0.0185 (11)	0.0149 (10)	−0.0033 (9)	0.0016 (8)	−0.0008 (8)
C12	0.0220 (11)	0.0175 (11)	0.0175 (10)	−0.0022 (9)	0.0015 (9)	0.0006 (9)
C13	0.0185 (10)	0.0190 (11)	0.0122 (9)	0.0023 (9)	−0.0007 (8)	0.0031 (8)
C14	0.0162 (10)	0.0187 (11)	0.0136 (10)	0.0009 (8)	−0.0033 (8)	−0.0002 (8)
C15	0.0147 (10)	0.0236 (12)	0.0223 (11)	0.0009 (9)	−0.0009 (8)	0.0004 (9)
C16	0.0154 (10)	0.0247 (12)	0.0199 (11)	0.0007 (9)	0.0042 (8)	−0.0008 (9)
C17	0.0139 (10)	0.0221 (11)	0.0105 (9)	−0.0023 (8)	0.0013 (7)	−0.0032 (8)
C18	0.0133 (9)	0.0148 (10)	0.0108 (9)	−0.0005 (8)	0.0031 (7)	0.0011 (7)
C19	0.0162 (10)	0.0174 (10)	0.0105 (9)	0.0012 (8)	−0.0003 (8)	−0.0005 (8)
C20	0.0229 (11)	0.0203 (11)	0.0092 (9)	0.0040 (9)	0.0023 (8)	−0.0007 (8)
C21	0.0161 (10)	0.0197 (11)	0.0121 (9)	0.0040 (8)	0.0034 (8)	0.0026 (8)
C22	0.0130 (9)	0.0147 (10)	0.0122 (9)	0.0015 (8)	0.0020 (7)	0.0027 (7)
C23	0.0143 (10)	0.0158 (10)	0.0110 (9)	−0.0008 (8)	0.0026 (7)	0.0007 (8)
C24	0.0130 (10)	0.0214 (11)	0.0128 (9)	−0.0018 (8)	0.0021 (7)	0.0032 (8)
C25	0.0125 (10)	0.0196 (11)	0.0150 (10)	0.0003 (8)	0.0021 (8)	0.0011 (8)
C26	0.0128 (10)	0.0153 (10)	0.0179 (10)	0.0017 (8)	0.0017 (8)	0.0021 (8)
C27	0.0104 (9)	0.0143 (10)	0.0185 (10)	0.0007 (8)	0.0026 (8)	0.0005 (8)
C28	0.0169 (11)	0.0162 (11)	0.0218 (11)	0.0006 (8)	0.0048 (8)	−0.0021 (9)
C29	0.0180 (11)	0.0148 (11)	0.0289 (12)	0.0002 (9)	0.0041 (9)	−0.0018 (9)
C30	0.0222 (12)	0.0162 (11)	0.0288 (12)	−0.0007 (9)	0.0066 (10)	0.0050 (9)
C31	0.0183 (11)	0.0184 (11)	0.0215 (11)	0.0012 (9)	0.0040 (9)	0.0050 (9)
C32	0.0122 (9)	0.0180 (11)	0.0157 (10)	−0.0035 (8)	−0.0013 (8)	0.0001 (8)
C33	0.0124 (9)	0.0175 (10)	0.0173 (10)	−0.0041 (8)	0.0026 (8)	−0.0021 (8)
C34	0.0144 (10)	0.0184 (11)	0.0247 (11)	−0.0029 (9)	0.0018 (9)	−0.0014 (9)
C35	0.0181 (11)	0.0199 (12)	0.0274 (12)	−0.0034 (9)	0.0061 (9)	−0.0089 (9)
C36	0.0210 (12)	0.0261 (13)	0.0195 (11)	−0.0054 (10)	0.0057 (9)	−0.0082 (9)
C37	0.0180 (11)	0.0229 (12)	0.0163 (10)	−0.0043 (9)	0.0023 (8)	−0.0034 (9)
C38	0.0129 (10)	0.0199 (11)	0.0154 (10)	−0.0041 (8)	0.0015 (8)	−0.0030 (8)
N1	0.0102 (8)	0.0178 (9)	0.0108 (8)	−0.0006 (7)	0.0018 (6)	−0.0019 (7)
N2	0.0120 (8)	0.0183 (9)	0.0125 (8)	−0.0030 (7)	0.0007 (6)	0.0013 (7)
O1	0.0190 (8)	0.0139 (7)	0.0141 (7)	0.0012 (6)	0.0042 (6)	−0.0003 (6)
O2	0.0192 (8)	0.0197 (8)	0.0130 (7)	0.0020 (6)	0.0017 (6)	−0.0014 (6)
Rh1	0.01149 (8)	0.01228 (8)	0.00945 (8)	−0.00042 (6)	0.00196 (5)	−0.00103 (6)
Rh2	0.01157 (8)	0.01501 (8)	0.00967 (8)	−0.00147 (6)	0.00068 (6)	−0.00002 (6)
C39	0.0268 (13)	0.0208 (12)	0.0216 (11)	0.0053 (10)	−0.0014 (9)	−0.0037 (9)
Cl1	0.0298 (3)	0.0381 (4)	0.0335 (3)	0.0075 (3)	−0.0045 (3)	−0.0158 (3)
Cl2	0.0229 (3)	0.0498 (4)	0.0269 (3)	0.0069 (3)	0.0051 (2)	−0.0056 (3)

### Geometric parameters (Å, º)

**Table:**

C1—C2	1.405 (3)	C17—H17B	0.99
C1—C8	1.519 (3)	C18—C19	1.395 (3)
C1—Rh1	2.121 (2)	C18—C23	1.397 (3)
C1—H1	0.95	C19—C20	1.393 (3)
C2—C3	1.526 (3)	C19—H19	0.95
C2—Rh1	2.132 (2)	C20—C21	1.386 (3)
C2—H2	0.95	C20—H20	0.95
C3—C4	1.538 (3)	C21—C22	1.397 (3)
C3—H3A	0.99	C21—H21	0.95
C3—H3B	0.99	C22—C23	1.390 (3)
C4—C5	1.512 (3)	C22—C24	1.516 (3)
C4—H4A	0.99	C23—H23	0.95
C4—H4B	0.99	C24—N2	1.482 (3)
C5—C6	1.394 (3)	C24—H24A	0.99
C5—Rh1	2.129 (2)	C24—H24B	0.99
C5—H5	0.95	C25—N1	1.296 (3)
C6—C7	1.523 (3)	C25—C26	1.434 (3)
C6—Rh1	2.132 (2)	C25—H25	0.95
C6—H6	0.95	C26—C31	1.420 (3)
C7—C8	1.541 (3)	C26—C27	1.434 (3)
C7—H7A	0.99	C27—O1	1.298 (3)
C7—H7B	0.99	C27—C28	1.422 (3)
C8—H8A	0.99	C28—C29	1.376 (3)
C8—H8B	0.99	C28—H28	0.95
C9—C10	1.408 (3)	C29—C30	1.413 (3)
C9—C16	1.524 (3)	C29—H29	0.95
C9—Rh2	2.138 (2)	C30—C31	1.368 (3)
C9—H9	0.95	C30—H30	0.95
C10—C11	1.516 (3)	C31—H31	0.95
C10—Rh2	2.102 (2)	C32—N2	1.297 (3)
C10—H10	0.95	C32—C33	1.442 (3)
C11—C12	1.540 (3)	C32—H32	0.95
C11—H11A	0.99	C33—C34	1.419 (3)
C11—H11B	0.99	C33—C38	1.425 (3)
C12—C13	1.515 (3)	C34—C35	1.375 (3)
C12—H12A	0.99	C34—H34	0.95
C12—H12B	0.99	C35—C36	1.411 (4)
C13—C14	1.388 (3)	C35—H35	0.95
C13—Rh2	2.139 (2)	C36—C37	1.380 (4)
C13—H13	0.95	C36—H36	0.95
C14—C15	1.517 (3)	C37—C38	1.425 (3)
C14—Rh2	2.136 (2)	C37—H37	0.95
C14—H14	0.95	C38—O2	1.298 (3)
C15—C16	1.540 (3)	N1—Rh1	2.0831 (18)
C15—H15A	0.99	N2—Rh2	2.0903 (19)
C15—H15B	0.99	O1—Rh1	2.0437 (16)
C16—H16A	0.99	O2—Rh2	2.0260 (16)
C16—H16B	0.99	C39—Cl1	1.768 (3)
C17—N1	1.482 (3)	C39—Cl2	1.768 (3)
C17—C18	1.522 (3)	C39—H39A	0.99
C17—H17A	0.99	C39—H39B	0.99
			
C2—C1—C8	125.3 (2)	C20—C19—H19	119.9
C2—C1—Rh1	71.13 (12)	C18—C19—H19	119.9
C8—C1—Rh1	110.28 (14)	C21—C20—C19	120.3 (2)
C2—C1—H1	117.4	C21—C20—H20	119.9
C8—C1—H1	117.4	C19—C20—H20	119.9
Rh1—C1—H1	88.6	C20—C21—C22	120.4 (2)
C1—C2—C3	123.0 (2)	C20—C21—H21	119.8
C1—C2—Rh1	70.28 (13)	C22—C21—H21	119.8
C3—C2—Rh1	113.35 (14)	C23—C22—C21	118.7 (2)
C1—C2—H2	118.5	C23—C22—C24	122.51 (19)
C3—C2—H2	118.5	C21—C22—C24	118.62 (19)
Rh1—C2—H2	86.5	C22—C23—C18	121.6 (2)
C2—C3—C4	112.42 (19)	C22—C23—H23	119.2
C2—C3—H3A	109.1	C18—C23—H23	119.2
C4—C3—H3A	109.1	N2—C24—C22	114.26 (17)
C2—C3—H3B	109.1	N2—C24—H24A	108.7
C4—C3—H3B	109.1	C22—C24—H24A	108.7
H3A—C3—H3B	107.9	N2—C24—H24B	108.7
C5—C4—C3	112.38 (18)	C22—C24—H24B	108.7
C5—C4—H4A	109.1	H24A—C24—H24B	107.6
C3—C4—H4A	109.1	N1—C25—C26	129.3 (2)
C5—C4—H4B	109.1	N1—C25—H25	115.4
C3—C4—H4B	109.1	C26—C25—H25	115.4
H4A—C4—H4B	107.9	C31—C26—C27	119.2 (2)
C6—C5—C4	124.8 (2)	C31—C26—C25	117.3 (2)
C6—C5—Rh1	71.03 (12)	C27—C26—C25	123.5 (2)
C4—C5—Rh1	111.38 (14)	O1—C27—C28	119.1 (2)
C6—C5—H5	117.6	O1—C27—C26	124.2 (2)
C4—C5—H5	117.6	C28—C27—C26	116.7 (2)
Rh1—C5—H5	87.6	C29—C28—C27	122.2 (2)
C5—C6—C7	123.7 (2)	C29—C28—H28	118.9
C5—C6—Rh1	70.76 (12)	C27—C28—H28	118.9
C7—C6—Rh1	113.52 (14)	C28—C29—C30	120.7 (2)
C5—C6—H6	118.2	C28—C29—H29	119.6
C7—C6—H6	118.2	C30—C29—H29	119.6
Rh1—C6—H6	85.8	C31—C30—C29	118.4 (2)
C6—C7—C8	111.54 (18)	C31—C30—H30	120.8
C6—C7—H7A	109.3	C29—C30—H30	120.8
C8—C7—H7A	109.3	C30—C31—C26	122.5 (2)
C6—C7—H7B	109.3	C30—C31—H31	118.8
C8—C7—H7B	109.3	C26—C31—H31	118.8
H7A—C7—H7B	108	N2—C32—C33	129.2 (2)
C1—C8—C7	112.93 (18)	N2—C32—H32	115.4
C1—C8—H8A	109	C33—C32—H32	115.4
C7—C8—H8A	109	C34—C33—C38	119.1 (2)
C1—C8—H8B	109	C34—C33—C32	117.2 (2)
C7—C8—H8B	109	C38—C33—C32	123.7 (2)
H8A—C8—H8B	107.8	C35—C34—C33	122.1 (2)
C10—C9—C16	123.6 (2)	C35—C34—H34	118.9
C10—C9—Rh2	69.24 (12)	C33—C34—H34	118.9
C16—C9—Rh2	113.92 (15)	C34—C35—C36	118.8 (2)
C10—C9—H9	118.2	C34—C35—H35	120.6
C16—C9—H9	118.2	C36—C35—H35	120.6
Rh2—C9—H9	86.9	C37—C36—C35	120.7 (2)
C9—C10—C11	124.7 (2)	C37—C36—H36	119.6
C9—C10—Rh2	71.98 (13)	C35—C36—H36	119.6
C11—C10—Rh2	109.97 (14)	C36—C37—C38	121.4 (2)
C9—C10—H10	117.6	C36—C37—H37	119.3
C11—C10—H10	117.6	C38—C37—H37	119.3
Rh2—C10—H10	88	O2—C38—C33	124.5 (2)
C10—C11—C12	112.72 (19)	O2—C38—C37	117.8 (2)
C10—C11—H11A	109	C33—C38—C37	117.8 (2)
C12—C11—H11A	109	C25—N1—C17	112.87 (18)
C10—C11—H11B	109	C25—N1—Rh1	122.51 (15)
C12—C11—H11B	109	C17—N1—Rh1	124.24 (15)
H11A—C11—H11B	107.8	C32—N2—C24	113.69 (19)
C13—C12—C11	111.59 (19)	C32—N2—Rh2	122.71 (15)
C13—C12—H12A	109.3	C24—N2—Rh2	123.52 (15)
C11—C12—H12A	109.3	C27—O1—Rh1	127.33 (14)
C13—C12—H12B	109.3	C38—O2—Rh2	128.69 (14)
C11—C12—H12B	109.3	O1—Rh1—N1	90.16 (7)
H12A—C12—H12B	108	O1—Rh1—C1	160.40 (8)
C14—C13—C12	125.2 (2)	N1—Rh1—C1	92.18 (8)
C14—C13—Rh2	70.94 (13)	O1—Rh1—C5	84.03 (7)
C12—C13—Rh2	112.91 (15)	N1—Rh1—C5	164.29 (8)
C14—C13—H13	117.4	C1—Rh1—C5	98.19 (8)
C12—C13—H13	117.4	O1—Rh1—C6	88.08 (8)
Rh2—C13—H13	86.1	N1—Rh1—C6	156.47 (8)
C13—C14—C15	125.2 (2)	C1—Rh1—C6	82.06 (9)
C13—C14—Rh2	71.15 (13)	C5—Rh1—C6	38.20 (9)
C15—C14—Rh2	110.83 (15)	O1—Rh1—C2	159.06 (7)
C13—C14—H14	117.4	N1—Rh1—C2	99.52 (8)
C15—C14—H14	117.4	C1—Rh1—C2	38.59 (8)
Rh2—C14—H14	88	C5—Rh1—C2	81.73 (8)
C14—C15—C16	111.77 (19)	C6—Rh1—C2	90.16 (9)
C14—C15—H15A	109.3	O2—Rh2—N2	90.54 (7)
C16—C15—H15A	109.3	O2—Rh2—C10	153.81 (8)
C14—C15—H15B	109.3	N2—Rh2—C10	94.83 (8)
C16—C15—H15B	109.3	O2—Rh2—C14	85.21 (8)
H15A—C15—H15B	107.9	N2—Rh2—C14	157.89 (8)
C9—C16—C15	111.17 (19)	C10—Rh2—C14	98.53 (9)
C9—C16—H16A	109.4	O2—Rh2—C9	163.81 (8)
C15—C16—H16A	109.4	N2—Rh2—C9	98.92 (8)
C9—C16—H16B	109.4	C10—Rh2—C9	38.78 (8)
C15—C16—H16B	109.4	C14—Rh2—C9	81.09 (9)
H16A—C16—H16B	108	O2—Rh2—C13	85.29 (8)
N1—C17—C18	111.75 (18)	N2—Rh2—C13	163.18 (8)
N1—C17—H17A	109.3	C10—Rh2—C13	82.20 (9)
C18—C17—H17A	109.3	C14—Rh2—C13	37.91 (9)
N1—C17—H17B	109.3	C9—Rh2—C13	89.14 (9)
C18—C17—H17B	109.3	Cl1—C39—Cl2	110.95 (14)
H17A—C17—H17B	107.9	Cl1—C39—H39A	109.4
C19—C18—C23	118.6 (2)	Cl2—C39—H39A	109.4
C19—C18—C17	120.20 (19)	Cl1—C39—H39B	109.4
C23—C18—C17	121.16 (18)	Cl2—C39—H39B	109.4
C20—C19—C18	120.3 (2)	H39A—C39—H39B	108
			
C8—C1—C2—C3	3.7 (3)	C25—N1—Rh1—C5	−79.9 (4)
Rh1—C1—C2—C3	105.6 (2)	C17—N1—Rh1—C5	107.7 (3)
C8—C1—C2—Rh1	−101.9 (2)	C25—N1—Rh1—C6	73.6 (3)
C1—C2—C3—C4	−93.4 (3)	C17—N1—Rh1—C6	−98.7 (2)
Rh1—C2—C3—C4	−12.5 (2)	C25—N1—Rh1—C2	−173.26 (17)
C2—C3—C4—C5	29.4 (3)	C17—N1—Rh1—C2	14.36 (17)
C3—C4—C5—C6	48.7 (3)	C2—C1—Rh1—O1	−160.82 (19)
C3—C4—C5—Rh1	−32.5 (2)	C8—C1—Rh1—O1	−39.2 (3)
C4—C5—C6—C7	2.6 (3)	C2—C1—Rh1—N1	102.58 (13)
Rh1—C5—C6—C7	106.0 (2)	C8—C1—Rh1—N1	−135.81 (15)
C4—C5—C6—Rh1	−103.3 (2)	C2—C1—Rh1—C5	−65.59 (14)
C5—C6—C7—C8	−93.9 (2)	C8—C1—Rh1—C5	56.03 (16)
Rh1—C6—C7—C8	−12.1 (2)	C2—C1—Rh1—C6	−100.34 (14)
C2—C1—C8—C7	46.4 (3)	C8—C1—Rh1—C6	21.28 (15)
Rh1—C1—C8—C7	−34.3 (2)	C8—C1—Rh1—C2	121.6 (2)
C6—C7—C8—C1	30.5 (3)	C6—C5—Rh1—O1	94.49 (14)
C16—C9—C10—C11	−3.4 (3)	C4—C5—Rh1—O1	−144.64 (17)
Rh2—C9—C10—C11	102.2 (2)	C6—C5—Rh1—N1	163.3 (3)
C16—C9—C10—Rh2	−105.6 (2)	C4—C5—Rh1—N1	−75.8 (3)
C9—C10—C11—C12	−45.4 (3)	C6—C5—Rh1—C1	−65.89 (14)
Rh2—C10—C11—C12	36.1 (2)	C4—C5—Rh1—C1	54.98 (17)
C10—C11—C12—C13	−31.3 (3)	C4—C5—Rh1—C6	120.9 (2)
C11—C12—C13—C14	93.8 (3)	C6—C5—Rh1—C2	−100.91 (14)
C11—C12—C13—Rh2	11.6 (2)	C4—C5—Rh1—C2	19.96 (16)
C12—C13—C14—C15	−2.4 (4)	C5—C6—Rh1—O1	−82.79 (13)
Rh2—C13—C14—C15	102.7 (2)	C7—C6—Rh1—O1	157.97 (17)
C12—C13—C14—Rh2	−105.1 (2)	C5—C6—Rh1—N1	−168.77 (18)
C13—C14—C15—C16	−44.8 (3)	C7—C6—Rh1—N1	72.0 (3)
Rh2—C14—C15—C16	36.3 (2)	C5—C6—Rh1—C1	114.19 (14)
C10—C9—C16—C15	96.1 (3)	C7—C6—Rh1—C1	−5.05 (16)
Rh2—C9—C16—C15	15.9 (2)	C7—C6—Rh1—C5	−119.2 (2)
C14—C15—C16—C9	−34.0 (3)	C5—C6—Rh1—C2	76.34 (14)
N1—C17—C18—C19	158.9 (2)	C7—C6—Rh1—C2	−42.90 (17)
N1—C17—C18—C23	−20.4 (3)	C1—C2—Rh1—O1	162.04 (18)
C23—C18—C19—C20	1.9 (3)	C3—C2—Rh1—O1	43.6 (3)
C17—C18—C19—C20	−177.5 (2)	C1—C2—Rh1—N1	−81.46 (13)
C18—C19—C20—C21	0.1 (4)	C3—C2—Rh1—N1	160.13 (16)
C19—C20—C21—C22	−1.9 (4)	C3—C2—Rh1—C1	−118.4 (2)
C20—C21—C22—C23	1.6 (3)	C1—C2—Rh1—C5	114.39 (14)
C20—C21—C22—C24	177.6 (2)	C3—C2—Rh1—C5	−4.02 (16)
C21—C22—C23—C18	0.5 (3)	C1—C2—Rh1—C6	76.99 (13)
C24—C22—C23—C18	−175.4 (2)	C3—C2—Rh1—C6	−41.41 (17)
C19—C18—C23—C22	−2.2 (3)	C38—O2—Rh2—N2	−8.29 (19)
C17—C18—C23—C22	177.2 (2)	C38—O2—Rh2—C10	93.8 (2)
C23—C22—C24—N2	−27.7 (3)	C38—O2—Rh2—C14	−166.6 (2)
C21—C22—C24—N2	156.5 (2)	C38—O2—Rh2—C9	−134.3 (3)
N1—C25—C26—C31	−168.7 (2)	C38—O2—Rh2—C13	155.4 (2)
N1—C25—C26—C27	11.2 (4)	C32—N2—Rh2—O2	3.22 (18)
C31—C26—C27—O1	175.1 (2)	C24—N2—Rh2—O2	−173.31 (17)
C25—C26—C27—O1	−4.7 (3)	C32—N2—Rh2—C10	−151.12 (18)
C31—C26—C27—C28	−4.8 (3)	C24—N2—Rh2—C10	32.36 (17)
C25—C26—C27—C28	175.3 (2)	C32—N2—Rh2—C14	81.8 (3)
O1—C27—C28—C29	−175.2 (2)	C24—N2—Rh2—C14	−94.8 (3)
C26—C27—C28—C29	4.8 (3)	C32—N2—Rh2—C9	170.02 (18)
C27—C28—C29—C30	−1.3 (4)	C24—N2—Rh2—C9	−6.50 (18)
C28—C29—C30—C31	−2.4 (4)	C32—N2—Rh2—C13	−72.1 (3)
C29—C30—C31—C26	2.2 (4)	C24—N2—Rh2—C13	111.3 (3)
C27—C26—C31—C30	1.4 (4)	C9—C10—Rh2—O2	160.62 (16)
C25—C26—C31—C30	−178.7 (2)	C11—C10—Rh2—O2	39.3 (3)
N2—C32—C33—C34	175.0 (2)	C9—C10—Rh2—N2	−98.24 (13)
N2—C32—C33—C38	−6.9 (4)	C11—C10—Rh2—N2	140.46 (15)
C38—C33—C34—C35	−0.1 (3)	C9—C10—Rh2—C14	64.10 (14)
C32—C33—C34—C35	178.0 (2)	C11—C10—Rh2—C14	−57.21 (17)
C33—C34—C35—C36	−0.1 (4)	C11—C10—Rh2—C9	−121.3 (2)
C34—C35—C36—C37	0.6 (4)	C9—C10—Rh2—C13	98.42 (14)
C35—C36—C37—C38	−1.0 (4)	C11—C10—Rh2—C13	−22.88 (16)
C34—C33—C38—O2	179.4 (2)	C13—C14—Rh2—O2	−88.47 (14)
C32—C33—C38—O2	1.3 (4)	C15—C14—Rh2—O2	150.06 (17)
C34—C33—C38—C37	−0.2 (3)	C13—C14—Rh2—N2	−168.04 (18)
C32—C33—C38—C37	−178.2 (2)	C15—C14—Rh2—N2	70.5 (3)
C36—C37—C38—O2	−178.9 (2)	C13—C14—Rh2—C10	65.42 (14)
C36—C37—C38—C33	0.7 (3)	C15—C14—Rh2—C10	−56.05 (18)
C26—C25—N1—C17	174.0 (2)	C13—C14—Rh2—C9	100.18 (14)
C26—C25—N1—Rh1	0.9 (3)	C15—C14—Rh2—C9	−21.28 (17)
C18—C17—N1—C25	−72.5 (2)	C15—C14—Rh2—C13	−121.5 (2)
C18—C17—N1—Rh1	100.54 (19)	C10—C9—Rh2—O2	−148.3 (3)
C33—C32—N2—C24	179.8 (2)	C16—C9—Rh2—O2	−29.7 (4)
C33—C32—N2—Rh2	3.0 (3)	C10—C9—Rh2—N2	86.60 (14)
C22—C24—N2—C32	83.3 (2)	C16—C9—Rh2—N2	−154.80 (16)
C22—C24—N2—Rh2	−99.9 (2)	C16—C9—Rh2—C10	118.6 (2)
C28—C27—O1—Rh1	167.14 (15)	C10—C9—Rh2—C14	−115.78 (14)
C26—C27—O1—Rh1	−12.8 (3)	C16—C9—Rh2—C14	2.82 (17)
C33—C38—O2—Rh2	7.2 (3)	C10—C9—Rh2—C13	−78.57 (14)
C37—C38—O2—Rh2	−173.22 (15)	C16—C9—Rh2—C13	40.03 (17)
C27—O1—Rh1—N1	18.17 (18)	C14—C13—Rh2—O2	88.26 (14)
C27—O1—Rh1—C1	−78.8 (3)	C12—C13—Rh2—O2	−150.67 (17)
C27—O1—Rh1—C5	−176.45 (19)	C14—C13—Rh2—N2	164.4 (2)
C27—O1—Rh1—C6	−138.36 (19)	C12—C13—Rh2—N2	−74.6 (3)
C27—O1—Rh1—C2	136.2 (2)	C14—C13—Rh2—C10	−114.81 (14)
C25—N1—Rh1—O1	−11.91 (17)	C12—C13—Rh2—C10	6.26 (16)
C17—N1—Rh1—O1	175.71 (16)	C12—C13—Rh2—C14	121.1 (2)
C25—N1—Rh1—C1	148.62 (18)	C14—C13—Rh2—C9	−76.52 (14)
C17—N1—Rh1—C1	−23.75 (17)	C12—C13—Rh2—C9	44.55 (17)
